# Maternal empowerment, feeding knowledge, and infant nutrition: Evidence from rural China

**DOI:** 10.7189/jogh.14.04094

**Published:** 2024-06-07

**Authors:** Yunwei Chen, Yian Guo, Yuju Wu, Alexis Medina, Huan Zhou, Gary L Darmstadt

**Affiliations:** 1 Department of Health Policy and Management, Gillings School of Global Public Health, University of North Carolina at Chapel Hill, Chapel Hill, North Carolina, USA; 2 Stanford Center on China’s Economy and Institutions, Stanford University, Stanford, California, USA; 3 Department of Health Behavior and Social Medicine, West China School of Public Health and West China Fourth Hospital, Sichuan University, Sichuan, China; 4 Department of Pediatrics, Stanford University School of Medicine, Stanford, California, USA

## Abstract

**Background:**

Maternal empowerment – the capacity to make decisions within households – is linked to better child feeding and nutritional outcomes, but few studies have considered the mediating role of caregiver knowledge. Further, existing literature centres primarily on the husband-wife dyad while overlooking grandmothers as important childcare decision-makers.

**Methods:**

We collected primary data through household surveys in 2019 and 2021 from 1190 households with infants zero to six months living in rural western China. We identified the primary and secondary caregivers for each infant and assessed their feeding knowledge and practices, as well as infant nutritional status. We constructed a maternal empowerment index using a seven-item decision-making questionnaire and examined the relationship between maternal empowerment in childcare and household decisions, caregivers’ feeding knowledge, and infant feeding practices and nutritional outcomes.

**Results:**

Mothers had significantly higher levels of feeding knowledge than secondary caregivers (most were grandmothers, 72.7%), with average knowledge scores of 5.4 vs. 4.1, respectively, out of 9. Mothers and secondary caregivers with higher levels of feeding knowledge had significantly higher exclusive breastfeeding rates by 13–15 percentage points (*P* < 0.01) and 11–13 percentage points (*P* < 0.01), respectively. The knowledge of secondary caregivers was even more strongly associated with not feeding formula (15 percentage points, *P* < 0.01). Mothers empowered to make childcare decisions were more likely to exclusively breastfeed (12–13 percentage points, *P* < 0.01), less likely to formula feed (9–10 percentage points, *P* < 0.05), and more likely to have children with higher Z-scores for length-for-age (0.32–0.33, *P* < 0.01) and weight-for-age (0.24–0.25, *P* < 0.05). Effects remained after controlling for maternal feeding knowledge.

**Conclusions:**

While mothers’ and grandmothers’ feeding knowledge was both important for optimal infant feeding, grandmothers’ knowledge was particularly critical for practicing exclusive breastfeeding. Given the disparity in feeding knowledge between the two caregivers, our study further shows that mothers empowered in childcare decision-making were more likely to exclusively breastfeed their infants. This implies that some mothers with adequate knowledge may not practice optimal feeding because of lower decision-making power. Overall, our study highlights the role of secondary caregivers (grandmothers) in infant care and suggests that future child nutritional interventions may benefit from involving secondary caregivers (grandmothers).

**Registration:**

Parent trial registration: ISRCTN16800789.

Maternal empowerment is linked to improved child nutrition and infant and young child feeding (IYCF) practices, particularly in the first 1000 days of a child’s life [1–4]. The focus of women’s empowerment is often on ‘intrahousehold decision-making power,’ with access to greater decision-making power within the household presumably leading to improved nutrition-related decisions for their children, thereby improving child health and nutrition [5]. Mothers throughout the developing world often face restricted opportunities to make decisions within the household that may reduce the welfare of women and children. Therefore, many nutrition-sensitive interventions, including the United Nations Scale-up Nutrition initiative, have adopted maternal empowerment approaches to improve child nutrition [6]. Many conditional cash-transfer programmes have also been transferring money to women, assuming that more resources in the hands of women increase their intrahousehold decision-making power and that when given the chance, women make better decisions than men for their children, resulting in improved health [5].

While maternal empowerment is recognised as important for child nutrition and growth, evidence on how it influences child nutrition is mixed. Some studies found that mothers with higher levels of empowerment in food purchasing, personal choice, and child health care are more likely to breastfeed exclusively and have children with better child growth [3]. However, a recent systematic review found that of the 1316 identified associations between maternal empowerment and child nutrition, the majority (1055) were not statistically significant [7]. Similarly, they found inconclusive evidence of relationships between maternal empowerment and pathway variables, such as IYCF practices [7].

The relationship between maternal empowerment and child nutrition assumes that women possess proper nutrition knowledge and that mothers are able to make optimal feeding decisions for children once empowered within the household. However, few studies have explicitly examined women’s feeding knowledge and controlled for it when estimating the association between maternal empowerment and child feeding and nutrition, potentially due to a heavy reliance in the literature on secondary data such as the Demographic and Health Surveys (DHS), which do not collect data on caregivers’ feeding knowledge [7].

Additionally, existing literature has mostly considered women’s decision-making power relative to their husbands, assuming that women are constrained by gender-based rules and most intrahousehold bargaining happens between spouses [5]. These studies, however, have neglected to consider that most women and children in non-western societies live in extended and multigenerational families where more experienced women, especially grandmothers, play a central role as advisors to mothers and caregivers of children [8]. In contrast, men play a limited role in day-to-day child nutrition in many countries in Asia, Africa, and Latin America [9]. Grandmothers in many cultural contexts hold authority in the household due to their age and experience, and often coach younger women on how to feed and care for their children and influence infant feeding practices [10]. Yet, the literature provides little empirical insight into the empowerment dynamics between caregivers across generations and how the dynamics between caregivers may influence IYCF practices and child nutrition.

Here we present evidence from western China, where both mothers and grandmothers often serve as child caregivers, to estimate whether improving maternal empowerment is associated with improved IYCF practices and infant nutrition in infants aged zero to six months. The objective of this study is to examine whether there is a difference in feeding knowledge between mothers and secondary caregivers (principally grandmothers) in rural China, and whether rural Chinese mothers with higher decision-making power within the household are more likely to exclusively breastfeed their infants and have infants with better nutrition and growth outcomes, conditional on the feeding knowledge of caregivers. Specifically, we seek to address the following questions: In addition to mothers, who takes care of infants (i.e. who are the secondary caregivers)? How does a mother’s feeding knowledge compare to those of secondary caregivers? To what extent does a mother’s and a secondary caregiver’s feeding knowledge matter in terms of optimal infant feeding and nutritional outcomes? Is maternal empowerment associated with exclusive breastfeeding behaviour and infant nutrition and growth after accounting for the feeding knowledge of caregivers?

## METHODS

### Study design and data collection

The data we used for this study are part of a randomised controlled intervention trial that aimed to improve child health and maternal well-being in western China [11]. Briefly, the programme recruited community health workers (CHWs) to deliver stage-based health and nutrition information to rural families with pregnant women and infants through monthly home visits with the assistance of a tablet-based mHealth system in order to promote optimal caregiving practices for marginalised young children in remote rural areas. The programme took place in over 100 rural townships across four nationally designated poverty counties in Sichuan Province, although the counties have since been removed from the national poverty list. In the experiment, pregnant women and caregivers of infants zero to six months of age were identified by lists of target families provided by local township health centres and by lists of pregnant women provided by county hospitals at baseline. A township-level randomised controlled trial was then conducted that randomly assigned a part of rural townships to implement the programme, with one CHW recruited per treatment township to deliver the intervention. More information about the programme design can be found in Chen et al. (2023) [11]. The programme included two waves of baseline surveys, since the first round of implementation was disrupted by the COVID-19 outbreak.

We used two waves of baseline data collected by the same survey team of trained enumerators through interviews with families with babies aged 0–6 months. Primary and secondary caregivers were identified for each infant and were interviewed separately. We collected detailed information on the infant, the two caregivers, and the family. The first wave of data was collected from November to December 2019 and the second wave was collected from July to August 2021. For both waves of data collection, we excluded urban townships and focused on larger rural townships with populations of at least 10 000 people. In 2019, we randomly sampled 80 townships, selecting 20 townships per county. In 2021, we expanded the survey areas to all 119 rural townships with populations of at least 10 000 people within the four counties. We relied on the local township health centres to recruit families, because township health centres in rural China have the responsibility of supervising health services for infants in their catchment areas, and the infant list managed by rural township health centres was also the list the local government uses to manage newborn infants, therefore reflecting the actual practice of the public health system. The survey teams also randomly checked in with local communities to identify infants missing from the list managed by local township health centres; however, we found limited cases from this source.

The two waves of the survey recruited 1632 families with infants aged 0–6 months. Given that 95% of primary caregivers were the infants’ mothers, we focused our analyses on mother-infant pairs (n = 1552). As our main research question rested on the mother’s empowerment within the household and her household bargaining with secondary caregivers regarding childcare, we excluded 338 families without secondary caregivers. We also excluded 24 observations missing key variables. Our final analytic sample contained 1190 families (n = 616 in 2019, n = 574 in 2021) with infants aged zero to six months with a mother and a secondary caregiver living together in the family.

### Infant feeding behaviour and nutritional outcomes

In accordance with World Health Organization (WHO) guidelines, appropriate feeding practice specifies that no foods or liquids other than breastmilk be given to infants during the first six months [12]. Following WHO guidance, we measured exclusive breastfeeding using a 24-hour dietary recall survey instrument about the different types of foods and liquids the infant ate the day before the survey [13]. We coded exclusive breastfeeding as a binary variable assigned as 1 if the infant did not consume any foods or liquids other than breastmilk the day before the interview, and 0 otherwise.

We also examined anthropometric measurements for each infant as indicators of nutritional status. These measurements included anaemia status, weight-for-age z-scores, and length-for-age z-scores. To assess anaemia status, trained nurses used a HemoCue Hb 201+ finger prick system (Hemocue, Inc, Angelholm, Sweden) to measure haemoglobin (Hb) concentrations. Since there is no internationally accepted Hb cutoff for infants under six months, we defined the presence of anaemia using the cutoff of Hb <110 g/L for children ages 6–59 months [14]. In each survey round, nurses also measured the length of each infant to a precision of 0.1 cm and the weight of each infant to a precision of 0.1 kg, following procedural guidelines recommended by the WHO [15]. Physical indicators of length and weight were used to construct infant growth standards: length-for-age z-scores and weight-for-age z-scores. Following internationally recognised cutoffs, we considered infants to be underweight if they were <2 standard deviations (SDs) of weight-for-age z-scores and stunted if they were <2 SDs of length-for-age z-scores [15].

Among the 1190 infants in our sample, 1125 (94.5%) completed weight measures, 1126 (94.6%) completed length measures, and 818 (68.7%) completed a Hb test. Among the 372 infants not tested for Hb, 215 (57.8%) were due to their young age (younger than 42 days) and perceived difficulty with the finger prick test; 124 (33.3%) infant caregivers explicitly objected to finger prick Hb testing; and 33 (8.9%) infants were tried but failed the test because they were crying, sick, uncooperative, or for other reasons. Among the 65 infants missing a measure of weight, 52 infants had caregiver objections to weight testing or failed the test due to uncooperative behaviour, and 13 infants had biologically implausible weight scores for their age (outside range −6 SDs and 6 SDs) and were dropped following WHO guidelines [15]. For length measures, 54 infants missed the test and 10 infants had biologically implausible scores.

### Maternal empowerment

To measure maternal empowerment, we administered a questionnaire on women’s ability to make decisions within the household [3,16]. We carefully reviewed existing literature and adapted commonly used decision-making questions to the context of rural China to develop a seven-item questionnaire. The questionnaire pertained to who within the household makes decisions over seven items, including three questions related to general household decision-making (1. what food to buy for the family; 2. whether they purchase major home goods; and 3. how to spend family income) and four questions pertaining to child-related decision-making (4. whether to exclusively breastfeed the infant; 5. what to feed the infant; 6. what to do if the infant is sick; and 7. how much to spend on the infant’s health care). The respondents chose between three choices (1. other family members alone; 2. joint decisions; and 3. respondents alone). While some studies coded the responses on a 3-point Likert scale, we followed Ewerling et al. to give equal weights for joint decisions and woman’s sole decision (coded as 1) as against a lower loading in other family member’s sole decision (coded as −1) [16]. We then used principal components analysis (PCA) to derive maternal empowerment scores. This approach relates observed measures on seven decision-making questions to the latent factor of maternal empowerment and suggests the number of factors that should be extracted from all the measures. Figure S1 in the **Online Supplementary Document** suggests that two latent factors should be extracted from seven measures of maternal empowerment. Based on the factor loadings of these measures on two latent factors, we defined two dimensions of maternal empowerment: household empowerment and infant care empowerment (Table S1 in the **Online Supplementary Document**). Empowerment scores were described as standard deviations, with negative scores as disempowering and positive scores as empowering [4,16].

### Feeding knowledge of primary and secondary caregivers

We administered a 9-item infant feeding knowledge test to mothers and secondary caregivers in the survey (Table S2 in the **Online Supplementary Document**). Each respondent received a knowledge score ranging from 0 to 9, depending on how many questions they answered correctly. Caregivers with scores below 60% (i.e. 0–5) were regarded as having inadequate infant feeding knowledge, while those with scores above 60% (i.e. 6–9) were considered to have higher infant feeding knowledge. We also used a 70% cut-off and found qualitatively similar findings (Tables S3–S7 in the **Online Supplementary Document**). A total of 890 secondary caregivers out of 1190 families were surveyed on feeding knowledge. The other secondary caregivers declined to participate, either because they were too busy or not interested. Many secondary caregivers, usually grandmothers with low education, also had difficulty understanding survey questions.

### Control variables

The control variables in the analysis included infant characteristics (age in months, sex, low birth weight, preterm birth), demographic and socioeconomic variables on mothers and secondary caregivers (mother’s age, education, and migration history, and the identity of the secondary caregiver), and household characteristics (family size, the number of siblings the infant has, and a household asset index). The household asset index was constructed using polychoric PCA based on whether the family owned or had access to running water, a flush toilet, a water heater, a washing machine, a computer, internet, a refrigerator, an air conditioner, a motorbike/motorcycle, and a car/truck [17].

### Data analysis

The key variables were summarised using means and SDs for continuous variables and counts and percentages for binary and categorical variables. We then modelled the relationship between maternal empowerment, the caregivers’ feeding knowledge, and infant feeding practices and nutritional outcomes using logistic models for binary outcomes and ordinary least squares models for continuous outcome variables. Regression models were adjusted for the specified control variables with standard errors clustered at the township level. We also included cohort and county fixed-effects into our analysis by incorporating dummy variables created for each cohort (2019 and 2021) and each of the four counties. This approach allows us to control for the unobserved differences between the two cohorts surveyed in different years, as well as the unobserved characteristics specific to each county.

## RESULTS

### Sample characteristics

Among the 1190 infants aged zero to six months in our sample, infants were equally distributed across the age range, with 430 (36.1%) aged <2 months, 376 (31.6%) aged 2–4 months, 384 (32.3%) aged 4–6 months; 621 (52.2%) were male (**Table 1**). Small proportions of infants had low birth weight (<2500 g, n = 56, 4.7%) or preterm birth (<37 weeks gestational age, n = 55, 4.6%). Most of the mothers were <30 years old (n = 813, 68.3%) and 507 (42.6%) reported their highest level of education as higher than high school. About half (n = 620, 52.1%) of the mothers married into a family from the same township, while 662 (55.6%) of the mothers had migrated within the most recent 2 years (**Table 1**).

**Table 1:** Descriptive statistics of demographic characteristics

	Sample size	N (%)/Mean (SD)*
**Characteristics of infants**		
Infant's age		
*<2 months*	1190	430 (36.1%)
*2*–*4 months*	1190	376 (31.6%)
*4*–*6 months*	1190	384 (32.3%)
The infant is a boy (0/1)	1190	621 (52.2%)
Low birth weight (<2500 g) (0/1)	1190	56 (4.7%)
Preterm birth (<37 weeks gestation) (0/1)	1190	55 (4.6%)
**Characteristics of mothers**		
Mother's age >30 (0/1)	1190	377 (31.7%)
Highest education is high school and higher (0/1)	1190	507 (42.6%)
Mother marries into this family from the same township (0/1)	1190	620 (52.1%)
Mother migrated out within the recent two years (0/1)	1190	662 (55.6%)
**Characteristics of secondary caregivers**		
Identity of secondary caregiver		
*Paternal grandmother*	1190	731 (61.4%)
*Maternal grandmother*	1190	134 (11.3%)
*Father*	1190	268 (22.5%)
*Others*	1190	57 (4.8%)
**Characteristics of households**		
Household size (excluding the infant)		
*≤3 members*	1190	259 (21.8%)
*4–6 members*	1190	880 (73.9%)
*≥7 members*	1190	51 (4.3%)
Siblings of the infant		
*No siblings*	1190	435 (36.6%)
*One sibling*	1190	645 (54.2%)
*Two or more siblings*	1190	110 (9.2%)
Household asset index (PCA index score)	1190	0.0 (1.0)

PCA – principal components analysis, SD – standard deviation

*Data are presented in n (%) for binary variables and mean (SD) for continuous variables.

### Identity of sample caregivers

Most of the secondary caregivers were grandmothers (n = 865, 72.7%) and 731 (61.4%) of secondary caregivers were paternal grandmothers, while 134 (11.3%) were maternal grandmothers (**Table 1**). Approximately 80% (n = 931) of families had four or more household members (**Table 1**).

### Infant feeding practices and nutritional outcomes

Approximately one-third (n = 425, 35.7%) of infants were exclusively breastfed (**Table 2**). While 1042 (87.6%) infants were fed breastmilk (non-exclusively), 490 (41.2%) and 536 (45.0%) consumed formula and water, respectively, and 100 (8.4%) were introduced to complementary soft or semi-soft food. Among the 818 infants who completed a Hb test, nearly half (n = 394, 48.2%) had a Hb level <110 g/L and would be considered anaemic based on standards for older infants 6–59 months of age (**Table 2**). Among the 1125 infants who completed weight and length measures, few were underweight (n = 20, 1.8%) or stunted (n = 41, 3.6%).

**Table 2:** Descriptive statistics of infant outcomes, caregiver knowledge, and maternal empowerment

	Sample Size	N (%)/Mean (SD)*
**Infant feeding practice**		
The infant was exclusively breastfed (0/1)	1190	425 (35.7%)
The infant was fed breastmilk (0/1)	1190	1042 (87.6%)
The infant was fed formula (0/1)	1190	490 (41.2%)
The infant was fed water (0/1)	1190	536 (45.0%)
The infant was introduced soft or semi-solid food (0/1)	1190	100 (8.4%)
**Infant nutritional outcomes**†		
The infant had anaemia (0/1)	818	394 (48.2%)
The infant was underweight (0/1)	1125	20 (1.8%)
The infant was stunted (0/1)	1126	41 (3.6%)
**Feeding knowledge of mothers and secondary caregivers**		
Feeding knowledge of mothers (score: 0–9)	1190	5.4 (1.5)
Feeding knowledge of secondary caregivers (score: 0–9)	890	4.1 (1.5)
Mother has higher feeding knowledge (score >60%, i.e. 6–9)	1190	579 (48.7%)
Secondary caregiver has higher feeding knowledge (score >60%, i.e. 6–9)	890	155 (17.4%)
**Mother's empowerment‡**		
Mother is empowered to make childcare decisions (0/1)	1190	1034 (86.9%)
Mother is empowered to make household decisions (0/1)	1190	708 (59.5%)

SD – standard deviation

*Data are presented in n (%) for binary variables and mean (SD) for continuous variables.

†The nutritional outcome variables were created using the WHO standards: anaemia was defined as having a haemoglobin level lower than 110 g/L. Infants were classified as underweight if score less than −2 standard deviation weight-for-age z-scores and stunted if scores less than −2 standard deviation length-for-age z-scores.

‡The two dimensions of maternal empowerment, childcare empowerment and household empowerment, were derived from the principal component analysis, with positive empowerment scores interpreted as empowering.

### Feeding knowledge of caregivers

**Figure 1:**
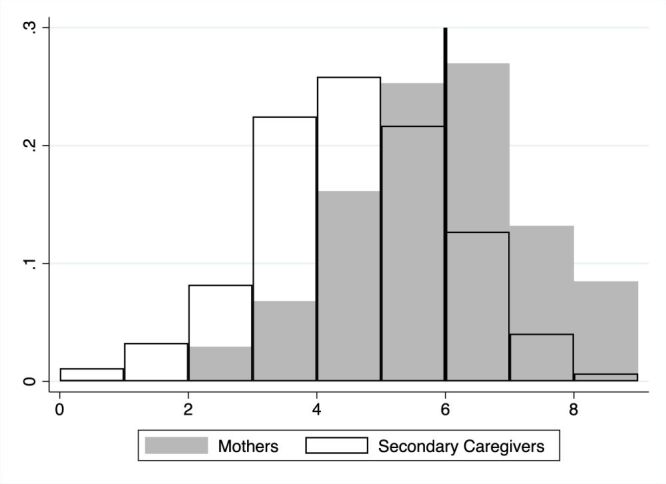
Comparison of infant feeding knowledge between mothers and secondary caregivers. Mothers and secondary caregivers were administered a 9-item infant feeding knowledge test. Each respondent received a knowledge score ranging from 0 to 9, depending on how many questions they answered correctly. The heavy vertical line at 6 on the x-axis represents the 60% cutoff we used to distinguish between caregivers with higher and lower feeding knowledge. Caregivers with scores below 60% (i.e. 0–5) were regarded as having inadequate infant feeding knowledge.

Among the 1190 mothers of infants in our sample, the average feeding knowledge score of mothers was 5.4 out of 9, and 579 (48.7%) mothers scored above 60% (i.e. 6–9) (**Table 2**). Secondary caregivers (n = 890), mostly grandmothers, who were successfully surveyed, had an average feeding knowledge score of 4.1, with only 155 (17.4%) scoring above 60%. Secondary caregivers had significantly lower levels of feeding knowledge than mothers (**Figure 1**).

### Maternal empowerment

The majority of mothers (n = 1034, 86.9%) were empowered to make childcare decisions and 708 (59.5%) were empowered to make household decisions (**Table 2**).

### Associations among caregiver feeding knowledge, maternal empowerment, and infant feeding practices

The feeding knowledge of both mothers and secondary caregivers, separately and together, was strongly correlated with increased rates of exclusive breastfeeding; the results are consistent after accounting for control variables and cohort and county fixed-effects (**Table 3**). High feeding knowledge among mothers was associated with increased exclusive breastfeeding rates by 13–15 percentage points (*P* < 0.01), and high feeding knowledge among secondary caregivers was associated with increased exclusive breastfeeding rates by 11–13 percentage points (*P* < 0.01). When controlling for maternal feeding knowledge, higher knowledge of the secondary caregiver was still significantly correlated with higher rates of exclusive breastfeeding with an increase of 11 percentage points (*P* < 0.01).

**Table 3:** Caregiver feeding knowledge and exclusive breastfeeding*

	Exclusive breastfeeding (yes = 1/no = 0)		
	(1)	(2)	(3)
Mother has high feeding knowledge (0/1)	0.15†		0.13†
	(0.03)		(0.03)
Secondary caregiver has high feeding knowledge (0/1)		0.13†	0.11†
		(0.04)	(0.04)
Observations	1190	890	890

*Data are average marginal effects with standard errors in parentheses and clustered at the township level. Estimation models are logistic models. All regressions control for cohort and county fixed-effects as well as infant, mother, secondary caregiver, and household characteristics (including infant age, gender, low birth weight or preterm delivery, mother's age, education, and migration history, identity of secondary caregivers, family structure, number of siblings, and family asset index).

†*P* < 0.01.

Logistic regression further showed that the feeding knowledge of both mothers and secondary caregivers was strongly correlated with the decreased probability of introducing formula, water, and solid or semi-solid food to infants (**Table 4**). Higher feeding knowledge of secondary caregivers showed a stronger correlation with not feeding formula to infants after controlling for maternal knowledge levels, with a decrease of 15 percentage points (*P* < 0.01).

**Table 4:** Caregiver feeding knowledge and introduction of complementary food*

	Feeding formula (yes = 1/no = 0)		Feeding water (yes = 1/no = 0)		Feeding solid or semi-solid food (yes = 1/no = 0)	
	(1)	(2)	(3)	(4)	(5)	(6)
Mother has high feeding knowledge (0/1)	−0.09†	−0.06	−0.13†	−0.10†	−0.04†	−0.04‡
	(0.03)	(0.03)	(0.02)	(0.03)	(0.01)	(0.02)
Secondary caregiver has high feeding knowledge (0/1)		−0.15†		−0.09‡		−0.04‡
		(0.04)		(0.04)		(0.02)
Observations	1190	890	1190	890	1190	890

*Data are average marginal effects with standard errors in parentheses and clustered at the township level. Estimation models are logistic models. All regressions control for cohort and county fixed-effects as well as infant, mother, secondary caregiver, and household characteristics (including infant age, gender, low birth weight or preterm delivery, mother's age, education, and migration history, identity of secondary caregivers, family structure, number of siblings, and family asset index). Columns 2, 4, and 6 additionally control for secondary caregiver's knowledge. For each dependent variable, we compare coefficients when secondary caregivers' knowledge is excluded or included in regressions.

†*P* < 0.01.

‡*P* < 0.05.

Mother’s childcare empowerment was strongly associated with increased exclusive breastfeeding (**Table 5**). Mothers who were empowered to make childcare decisions had a higher probability of exclusive breastfeeding with an increase of 12–13 percentage points (*P* < 0.01); the results were consistent after controlling for mothers’ feeding knowledge. Similarly, higher empowerment of mothers in childcare decision-making was associated with the decreased probability of formula feeding by 9–10 percentage points (*P* < 0.05). Meanwhile, no significant association was found between mother’s household empowerment and exclusive breastfeeding behaviour or formula feeding.

**Table 5:** Maternal empowerment and infant feeding*

	Exclusive breastfeeding (yes = 1/no = 0)		Formula feeding (yes = 1/no = 0)	
	(1)	(2)	(3)	(4)
Mother is empowered to make childcare decisions (0/1)	0.13†	0.12†	−0.10‡	−0.09‡
	(0.04)	(0.04)	(0.05)	(0.05)
Mother is empowered to make household decisions (0/1)	0.04	0.02	−0.01	−0.01
	(0.03)	(0.03)	(0.03)	(0.03)
Mother has high feeding knowledge (0/1)		0.14†		−0.08†
		(0.03)		(0.03)
Observations	1190	1190	1190	1190

*Data are average marginal effects with standard errors in parentheses and clustered at the township level. Standard errors are in parentheses and clustered at the township level. Estimation models are logistic models. All regressions control for cohort and county fixed-effects as well as infant, mother, secondary caregiver, and household characteristics (including infant age, gender, low birth weight or preterm delivery, mother's age, education, and migration history, identity of secondary caregivers, family structure, number of siblings, and family asset index). Columns 2 and 4 additionally control for maternal knowledge. For each dependent variable, we compare coefficients when maternal knowledge is excluded or included in regressions.

†*P* < 0.01.

‡*P* < 0.05.

### Associations among caregiver feeding knowledge, maternal empowerment, and infant nutritional outcomes

Caregiver feeding knowledge did not show strong correlations with any of the infant nutritional outcomes we assessed in this analysis (**Table 6**). However, mothers empowered to make childcare decisions were more likely to have children with 0.32–0.33 higher z-scores in length-for-age (*P* < 0.01) and 0.24–0.25 higher z-scores in weight-for-age (*P* < 0.05). The effects are consistent after controlling for mothers’ feeding knowledge (**Table 7**). Anaemia did not show similar correlations with mothers’ empowerment.

**Table 6:** Caregiver knowledge and infant nutritional outcomes*

	Anaemia (yes = 1/no = 1)		Length-for-age (z-score)		Weight-for-age (z-score)	
	(1)	(2)	(3)	(4)	(5)	(6)
Mother has high feeding knowledge (0/1)	−0.05	−0.07	0.07	0.14	0.09	0.12
	(0.04)	(0.05)	(0.09)	(0.09)	(0.07)	(0.08)
Secondary caregiver has high feeding knowledge (0/1)		0.02		0.03		0.18
		(0.05)		(0.09)		(0.09)
Observations	818	619	1126	852	1125	850

*Data are average marginal effects with standard errors in parentheses and clustered at the township level. Estimation models are logistic models for binary outcomes and OLS models for continuous outcomes. All regressions control for whether the infant was exclusively breastfed, cohort and county fixed-effects as well as infant, mother, secondary caregiver, and household characteristics (including infant age, gender, low birth weight or preterm delivery, mother's age, education, and migration history, identity of secondary caregivers, family structure, number of siblings, and family asset index). Columns 2, 4, and 6 additionally control for secondary caregiver's knowledge. For each dependent variable, we compare coefficients when secondary caregivers' knowledge is excluded or included in regressions.

**Table 7:** Mother’s empowerment and infant nutritional outcomes*

	Anaemia (yes = 1/no = 0)		Length-for-age (z-score)		Weight-for-age (z-score)	
	(1)	(2)	(3)	(4)	(5)	(6)
Mother is empowered to make childcare decisions (0/1)	0.03	0.04	0.33†	0.32†	0.25‡	0.24‡
	(0.05)	(0.05)	(0.11)	(0.11)	(0.12)	(0.12)
Mother is empowered to make household decisions (0/1)	−0.02	−0.01	0.05	0.05	0.11	0.11
	(0.03)	(0.03)	(0.07)	(0.07)	(0.08)	(0.07)
Mother has high feeding knowledge (0/1)		−0.05		0.05		0.06
		(0.04)		(0.09)		(0.07)
Observations	818	818	1126	1126	1125	1125

## DISCUSSION

This study presents evidence from western China on the association between maternal empowerment, maternal and secondary caregiver knowledge regarding infant nutrition and their IYCF practices. Infant feeding practices were generally poor in our sample: only about one-third of infants younger than 6 months of age were exclusively breastfed while nearly half received formula, in line with national assessments of exclusive breastfeeding rates in China [18]. Nearly half of the sampled infants were anaemic (48.2%, based on anaemia assessment standards for infants 6–59 months of age) although few (<4%) were stunted or underweight. These nutritional statistics indicate that young children in our study area have similar nutritional deficiencies as their counterparts across rural China; anaemia prevalence among young children living in rural areas has consistently been found to be high (40–55%) while stunting and underweight rates are 2–5 and 2%, respectively [19–21]. Similarly, the proportion of infants with low birth weight (4.7%) was low in our sample, compared to a national rate of around 5.5% between 2012 and 2018 [22].

Approximately three-fourths (72.7%) of secondary caregivers were grandmothers, with the majority being paternal grandmothers (61.4%). This result is in line with evidence from Africa, Asia, and Latin America, and highlights the crucial yet often-overlooked role that grandmothers play in childcare in non-western societies [8,9]. Given that approximately 80% of families were extended and multigenerational with four or more family members, an infant’s father seemed to play a limited role in infant care.

We found that secondary caregivers had significantly lower levels of feeding knowledge than mothers. This disparity might be related to the differences in educational level and access to childcaring information through modern means such as the internet, as grandmothers mostly have lower education and are largely influenced by local culture and tradition [10]. Previous research supports this finding that grandmothers have significantly lower feeding knowledge than mothers [23], which is concerning since grandmothers are often advisors to young mothers due to their age and experience [9]. Poor secondary caregiver knowledge may be problematic, especially in China, since many mothers out-migrate as their children grow older, leaving less knowledgeable secondary caregivers to raise children [24].

While previous nutrition interventions often targeted mothers [25,26], our study showed that higher feeding knowledge of both mothers and secondary caregivers was strongly correlated with increased optimal infant feeding, highlighting the role of secondary caregivers in practicing optimal infant feeding. Higher feeding knowledge of secondary caregivers, mostly grandmothers, was significantly associated with exclusive breastfeeding and not giving water and complementary foods too early, even after controlling for mother’s knowledge. In particular, feeding knowledge of secondary caregivers mattered more in not feeding formula to infants, compared to that of mothers. This finding confirms previous research showing that grandmothers’ correct knowledge translates into optimal IYCF practices [23]. One possible explanation is that grandmothers, who tend to follow local norms rather than national/international recommended practices, may exert a strong influence on young mothers and infant feeding due to their age and experience. For example, one study found that in China, there is a common misconception that breastmilk is less nutritious than formula, and mothers with this misconception reported learning it from older female family members [27]. Grandmothers may be particularly influential when they come from the paternal side of the family, as was most common in our study population; mothers in these contexts may be hard-pressed to resist the grandmothers’ wishes. These results may suggest the importance of improving the feeding knowledge of secondary caregivers, in addition to that of primary caregivers, in future child nutrition interventions. Institutions such as UNICEF, the World Bank, and WHO are already in support of grandmother-involved childcare programmes and interventions [28].

In our main analysis examining maternal empowerment, we found that mothers with higher participation in childcare decision-making (for example, what to feed the infant, what to do if the infant is sick, etc.) were more likely to exclusively breastfeed their infants and less likely to engage in formula feeding after accounting for feeding knowledge. However, mother’s household empowerment (e.g. what food to buy for the family, how to spend family income, etc.) was not significantly correlated with exclusive breastfeeding behaviour, perhaps because our sample included infants of a younger age. Unlike many previous studies that have examined nutritional outcomes in older children [7], we focused on infants aged 0–6 months who are predominantly breastfed and may not directly benefit from family resource allocation. These results are in line with previous research suggesting that different dimensions of maternal empowerment may relate to family health behaviour in different ways [3]. Even so, the significant associations between maternal childcare empowerment and optimal infant feeding confirm some of the prior research that maternal empowerment affects optimal caregiving [1,2]. These results suggest that some daughters and daughters-in-law, despite having a higher level of feeding knowledge, may not be able to translate that knowledge into optimal infant feeding practices due to relatively lower decision-making power or bargaining power in child-care decisions [9]. Though grandmothers’ knowledge is often not up to date, they possibly wield significant influence over both young mothers and infant care, due to their experience and authority within the household. These results further confirm that mother-only nutritional interventions may be inadequate, and it may be necessary to involve secondary caregivers (especially grandmothers) in future interventions.

In contrast to optimal infant feeding practices, infant nutritional outcomes were not clearly linked with maternal empowerment and caregiver knowledge. While higher levels of feeding knowledge of the two caregivers did not seem to translate into better nutritional outcomes for the infant, mothers with higher participation in childcare decision-making had infants with higher length and weight for their age. Anaemia was not linked with maternal empowerment or caregiver knowledge. These paradoxical findings may reflect our sample of infants, who may have been too young for improved caregiver feeding knowledge and practices to produce clear benefits. This does not mean, however, that young children would not benefit from improved maternal feeding knowledge and practices in the long run. Several studies have documented that child health in early childhood and the benefits of better caregiving behaviours can last throughout children’s lives and affect their health and well-being at later ages [29]. Additionally, mother’s childcare empowerment may affect infant growth through mechanisms other than exclusive breastfeeding. Although exclusive breastfeeding is strongly correlated with caregiver feeding knowledge and maternal empowerment, the links between breastfeeding and infant anaemia and nutrition may be weak. Bhutta et al. reviewed interventions that affect child nutrition-related outcomes and found that strategies for breastfeeding promotion often have a larger effect on survival but limited effects on infant nutrition [26]. This may also explain why exclusive breastfeeding does not show a significant correlation with infant nutritional outcomes in our analysis. Lastly, the smaller sample size of infants for whom Hb measures were available, reflecting the difficulty in obtaining these samples in young infants, may have undermined the power of the analysis to find significant results between anaemia status with maternal empowerment and knowledge.

Our study builds on previous studies and contributes to the literature in several ways. First, we presented evidence from rural China and focused on the decision-making dynamic on infant feeding between mothers and secondary caregivers, mostly grandmothers. Understanding the role of maternal empowerment in extended families and its relationship with child feeding and nutrition has important implications for other countries with similar cultural settings [30]. Second, we controlled for feeding knowledge of mothers and secondary caregivers, since improving maternal empowerment does not necessarily lead to optimal household decision-making for children unless mothers possess appropriate child caring knowledge compared to other caregivers in the household. We compared the feeding knowledge of mothers and secondary caregivers to examine the potential role of caregiver knowledge as a mediating factor between maternal empowerment and infant nutritional outcomes. Third, most previous studies examined growth outcomes in older children [7], while we focused on infants aged 0–6 months, a critical period when maternal empowerment could influence an infant’s nutrition through the practice of breastfeeding. Hence, in addition to the nutritional outcomes that have been commonly examined in the literature, we also included exclusive breastfeeding in our study as a critical and modifiable feeding practice. Finally, there is a lack of evidence on how maternal empowerment is associated with child nutrition and optimal child feeding in the context of China, since most existing studies of women’s empowerment have been conducted in South Asia and sub-Saharan Africa [7]. Anaemia affects 40–55% of young children in rural China and appears to be largely attributable to inadequate caregiver feeding knowledge and practice [18,19,21]. Many rural families overemphasise formula feeding and diminish breastfeeding due to the misconception that breastmilk is less nutrient-dense than formula [27]. This poor feeding pattern suggests that mother’s knowledge and empowerment could significantly influence an infant’s nutrition through the practice of breastfeeding.

We acknowledge several limitations of this study. First, we only tested two domains of empowerment in our sample area (household decision-making and childcare decision-making), while Santoso et al. suggested other indicators that could be crucial for infant feeding and nutrition, such as time use and resources [7]. Considering that maternal empowerment can be context-specific, future studies may consider testing more domains of empowerment in more areas of China. Second, because of the cross-sectional and observational nature of our data, we lacked the ability to make causal inferences between maternal empowerment and infant feeding and nutrition. However, given that this study is the first study (or one of the few studies) to estimate this relationship in the context of China, the strong correlation presented in this study is informative for future research to be conducted in China. Third, only 890 secondary caregivers out of 1190 families were surveyed on feeding knowledge, and the rest refused to participate. As a result of this missing feeding knowledge data from secondary caregivers, the regression models that included it may have produced biased results. To evaluate possible bias, we compared the coefficients across the full sample (n = 1190) and the restricted sample (n = 890), including and excluding secondary caregiver feeding knowledge in most specifications, and found no significant differences. Lastly, this study pooled cross-sectional data from two infant cohorts surveyed in the same region in different periods (2019 and 2021) to increase the power of the analysis. We acknowledge that the COVID pandemic that started in early 2020 may have affected family caregiving behaviour and infant health in unobserved ways. However, the effect of the COVID pandemic was not the focus of this study, so we included a cohort (wave) fixed effect to control for any unobserved differences between the two cohorts surveyed from different years, including those caused by the pandemic.

## CONCLUSIONS

In conclusion, we examined the association between maternal empowerment and infant feeding and nutrition in a rural setting in western China. Our analysis shows that maternal empowerment in childcare decision-making can influence the practice of exclusive breastfeeding for infants younger than 6 months and produce better infant growth outcomes. Decision-making dynamics regarding childcare occur between mothers and secondary caregivers, most of whom are grandmothers rather than fathers, as assumed in many previous studies. Although grandmothers’ knowledge is often not up-to-date, grandmothers can exert significant influence over both young mothers and infant care due to their authority within the household. Our findings highlight that involving secondary caregivers in future child nutrition interventions may be essential, especially in non-western societies where multigenerational families are common.

## Additional material


Online Supplementary Document
