# Supplementary material for: Maternal empowerment, feeding knowledge, and infant nutrition: Evidence from rural China
Source: J Glob Health. 2024 Jun 7;14:04094. doi: 10.7189/jogh.14.04094 (PMC11157471; doi:10.7189/jogh.14.04094)
Supplement: Online Supplementary Document [file jogh-14-04094-s001.pdf]

Maternal Empowerment, Feeding Knowledge, and Infant Nutrition: Evidence from Rural China

Online Supplementary Materials

Figure S1. Scree plot of eigenvalues of PCA for mother's empowerment.

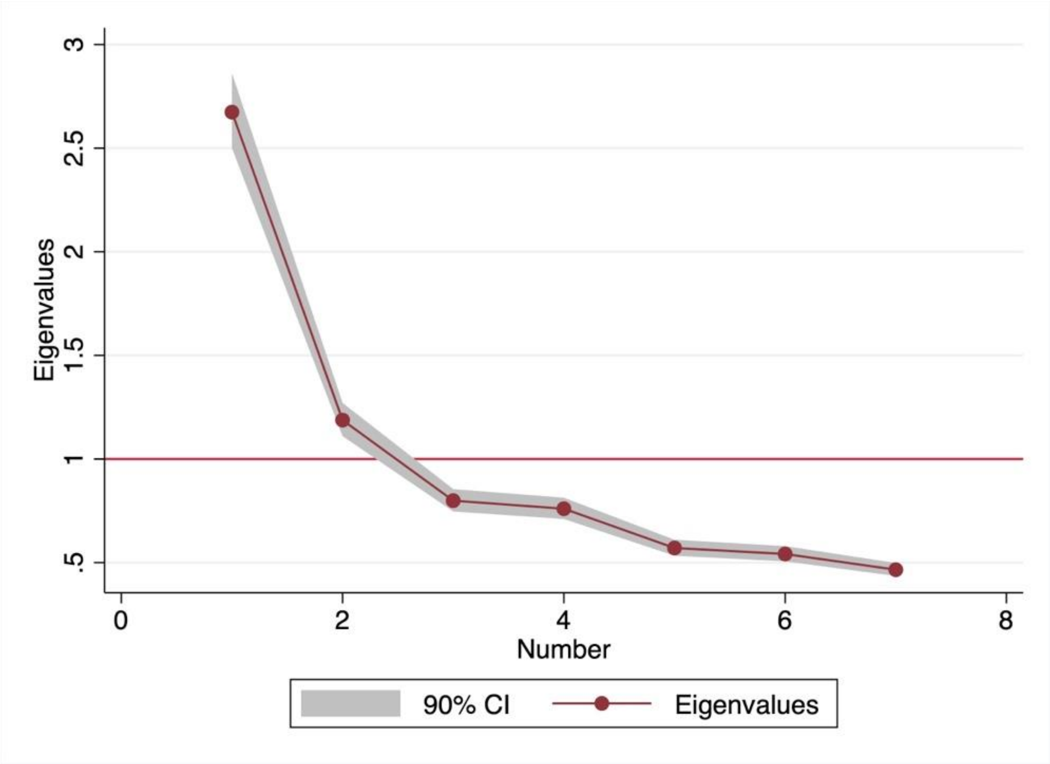

Table S1. Principal Component Analysis (PCA) factor loading of decision-making domains*

	**Childcare Empowerment**	**Household Empowerment**
What food to buy for the family	0.2655	0.5299
Whether to purchase major home goods	0.3401	0.4726
How to spend family income	0.4080	0.3690
Exclusive breastfeeding	0.3028	-0.3885
What food to feed the baby	0.4183	-0.3207
What to do if the child is sick	0.4406	-0.2493
How much to spend on infant's healthcare	0.4321	-0.2091

*Empowerment index is created based on the questions related to who makes decisions in the households. We follow Ewerling et al. (2020) for the coding: other family members alone = -1; joint decision or respondent alone=1.

Table S2. Caregiver feeding knowledge questionnaire

Questions	Responses
1. The first milk after birth is bad for your infant and should be discarded. Do you think it's right?	1=Correct;<br>**2=Incorrect;**<br>999=Don't Know
2. Women with small breasts cannot produce enough milk to feed an infant. Do you think it's right?	1=Correct;<br>**2=Incorrect;**<br>999=Don't Know
3. It is important to feed your infant water in addition to breastmilk. Do you think it's right?	1=Correct;<br>**2=Incorrect;**<br>999=Don't Know
4. Breastfeeding mothers should drink more than usual in order to ensure a good milk supply. Do you think it's right?	**1=Correct**;<br>2=Incorrect;<br>999=Don't Know
5. When should you breastfeed your infant?	1=Right before you have a meal<br>2=Right after you finish a meal<br>**3=Whenever he/she is hungry**<br>4=Every three hours, no matter what<br>999=Don't Know
6. When should you start to introduce water to your infant?	1=Immediately<br>2=Within the first month<br>**3=When he starts to eat solid foods**<br>4=When he starts to crawl<br>999=Don't Know
7. When should you start to introduce formula to your infant?	1=Around 3 months old<br>2=Around 6 months old<br>3=Around 1 year old<br>**4=Most babies don't need to ever drink formula**<br>999=Don't know
8. When should you start to introduce soft or semi-solid foods to your child?	1=Right away<br>**2=Around 6 months old**<br>3=Around 1 year old<br>4=Around 18 months old<br>999=Don't Know
9. What is the best way to know if your child has anemia?	1= The infant looks skinnier than other infants at his age<br>2=The infant's hair color looks light<br>**3=Blood test from doctor**<br>4=The infant has trouble breathing<br>999=Don't Know

Note: The correct answers are in bold.

Table S3. Caregiver feeding knowledge and exclusive breastfeeding (knowledge cutoff for defining high feeding knowledge = 70%)*

	Exclusive Breastfeeding (Yes=1 / No=0)		
	(1)	(2)	(3)
Mother has high feeding knowledge (0/1)	0.20***		0.19***
	(0.03)		(0.04)
Secondary caregiver has high feeding knowledge (0/1)		0.25***	0.19**
		(0.08)	(0.09)
Observations	1190	890	890

*Data are average marginal effects with standard errors are in parentheses and clustered at the township level. Estimation models are logistic models. All regressions control for cohort and county fixed-effects as well as infant, mother, secondary caregiver, and household characteristics (including infant age, gender, low birth weight or preterm delivery, mother's age, education, and migration history, identity of secondary caregivers, family structure, number of siblings, and family asset index). ** $p<0.05$ *** $p<0.01$

Table S4. Caregiver feeding knowledge and introduction of complementary food (knowledge cutoff for defining high feeding knowledge = 70%)*

	Formula (Yes=1 / No=0)		Water (Yes=1 / No=0)		Solid or Semi-solid Food (Yes=1 / No=0)	
	(1)	(2)	(3)	(4)	(5)	(6)
Mother has high feeding knowledge (0/1)	-0.10***	-0.08**	-0.23***	-0.22***	-0.03	-0.00
	(0.03)	(0.04)	(0.03)	(0.04)	(0.01)	(0.02)
Secondary caregiver has high feeding knowledge (0/1)		-0.27***		-0.19**		-0.03
		(0.06)		(0.08)		(0.03)
Observations	1190	890	1190	890	1190	890

*Standard errors are in parentheses and clustered at the township level. Estimation models are logistic models. All regressions control for cohort and county fixed-effects as well as infant, mother, secondary caregiver, and household characteristics (including infant age, gender, low birth weight or preterm delivery, mother's age, education, and migration history, identity of secondary caregivers, family structure, number of siblings, and family asset index). ** $p<0.05$ *** $p<0.01$

Table S5. Mother's empowerment and infant feeding (knowledge cutoff for defining high feeding knowledge = 70%)*

	Exclusive Breastfeeding (Yes=1 / No=0)		Formula Feeding (Yes=1 / No=0)	
	(1)	(2)	(3)	(4)
Mother is empowered to make childcare decisions (0/1)	0.13***	0.12***	-0.10**	-0.09**
	(0.04)	(0.04)	(0.05)	(0.05)
Mother is empowered to make household decisions (0/1)	0.04	0.03	-0.01	-0.01
	(0.03)	(0.03)	(0.03)	(0.03)
Mother has high feeding knowledge (0/1)		0.19***		-0.09***
		(0.03)		(0.03)
Observations	1190	1190	1190	1190

*Standard errors are in parentheses and clustered at the township level. Estimation models are logistic models. All regressions control for cohort and county fixed-effects as well as infant, mother, secondary caregiver, and household characteristics (including infant age, gender, low birth weight or preterm delivery, mother's age, education, and migration history, identity of secondary caregivers, family structure, number of siblings, and family asset index). ** $p<0.05$ *** $p<0.01$

Table S6. Caregiver knowledge and infant nutritional outcomes (knowledge cutoff for defining high feeding knowledge = 70%)*

	Anemia (0/1)		Height-for-age (z-score)		Weight-for-age (z-score)	
	(1)	(2)	(3)	(4)	(5)	(6)
Mother has high feeding knowledge (0/1)	0.05	0.03	0.07	0.05	0.10	0.06
	(0.05)	(0.06)	(0.11)	(0.11)	(0.08)	(0.10)
Secondary caregiver has high feeding knowledge (0/1)		-0.10		0.10		0.27
		(0.09)		(0.17)		(0.16)
Observations	818	619	1126	852	1125	850

*Standard errors are in parentheses and clustered at the township level. Estimation model for anemia is the logistic model and the estimation models for height-for-age and weight-for-age are OLS models. All regressions control for whether the infant was exclusively breastfed, cohort and county fixed-effects as well as infant, mother, secondary caregiver, and household characteristics (including infant age, gender, low birth weight or preterm delivery, mother's age, education, and migration history, identity of secondary caregivers, family structure, number of siblings, and family asset index). ** $p<0.05$ *** $p<0.01$

Table S7. Mother’s empowerment and infant nutritional outcomes (knowledge cutoff for defining high feeding knowledge = 70%)*

	Anemia (Yes=1 / No=0)		Height-for-age (z-score)		Weight-for-age (z-score)	
	(1)	(2)	(3)	(4)	(5)	(6)
Mother is empowered to make childcare decisions (0/1)	0.03	0.03	0.33***	0.32***	0.25**	0.24**
	(0.05)	(0.05)	(0.11)	(0.11)	(0.12)	(0.12)
Mother is empowered to make household decisions (0/1)	-0.02	-0.02	0.05	0.05	0.11	0.11
	(0.03)	(0.03)	(0.07)	(0.07)	(0.08)	(0.08)
Mother has high feeding knowledge (0/1)		0.05		0.05		0.09
		(0.05)		(0.11)		(0.09)
Observations	818	818	1126	1126	1125	1125

*Standard errors are in parentheses and clustered at the township level. Estimation model for anemia is logistic model and estimation models for height-for-age and weight-for-age are OLS models. All regressions control for whether the infant was exclusively breastfed, cohort and county fixed-effects as well as infant, mother, secondary caregiver, and household characteristics (including infant age, gender, low birth weight or preterm delivery, mother's age, education, and migration history, identity of secondary caregivers, family structure, number of siblings, and family asset index). ** p<0.05 *** p<0.01
